# Metabolic syndrome is associated with better quality of sleep in the oldest old: results from the “Mugello Study”

**DOI:** 10.1186/s13098-020-00554-y

**Published:** 2020-05-24

**Authors:** Alice Laudisio, Silvia Giovannini, Panaiotis Finamore, Luca Navarini, Domenico Paolo Emanuele Margiotta, Federica Vannetti, Claudio Macchi, Daniele Coraci, Isabella Imbimbo, Raffaello Molino-Lova, Claudia Loreti, Raffaele Antonelli Incalzi, Giuseppe Zuccalà, Luca Padua, the Mugello Study Working Group, Guglielmo Bonaccorsi, Roberta Boni, Chiara CastagnolI, Francesca Cecchi, Francesca Cesari, Francesco Epifani, Roberta Frandi, Betti Giusti, Maria Luisa Eliana Luisi, Rossella Marcucci, Raffaello Molino-Lova, Anita Paperini, Lorenzo Razzolini, Francesco Sofi, Nona Turcan, Debora Valecchi

**Affiliations:** 1 Unit of Geriatrics, Department of Medicine, Campus Bio-Medico di Roma University, Rome, Italy; 2 Department of Geriatrics, Neurosciences and Orthopaedics, Fondazione Policlinico Universitario A. Gemelli IRCCS, Rome, Italy; 3 Unit of Allergology, Immunology and Rheumatology, Department of Medicine, Università Campus Bio-Medico di Roma, Rome, Italy; 4 IRCCS Fondazione Don Carlo Gnocchi, Florence, Italy; 5 Università Cattolica del Sacro Cuore, Rome, Italy

**Keywords:** Metabolic syndrome, Sleep quality, Geriatric, Reverse epidemiology

## Abstract

**Background and aims:**

Reduced sleep quality is common in advanced age. Poor sleep quality is associated with adverse outcomes, chiefly cardiovascular, in young and middle-aged subjects, possibly because of its association with metabolic syndrome (MetS). However, the correlates of sleep quality in oldest populations are unknown. We evaluated the association of sleep quality with MetS in a cohort of subjects aged 90+.

**Methods and results:**

We analysed data of 343 subjects aged 90+ living in the Mugello area (Tuscany, Italy). Quality of sleep was assessed using the Pittsburgh Sleep Quality Assessment Index (PSQI). Good quality of sleep was defined by a PSQI score < 5. MetS was diagnosed according to the National Cholesterol Education Program’s ATP-III criteria; 83 (24%) participants reported good quality of sleep. MetS was diagnosed in 110 (24%) participants. In linear and logistic models, MetS was inversely associated with PSQI score ((B = − 1.04; 95% CI − 2.06 to − .03; P = .044), with increased probability of good sleep quality (OR = 2.52; 95% CI 1.26–5.02; P = .009), and with a PSQI below the median (OR = 2.11; 95% CI 1.11–3.40, P = .022), after adjusting. None of the single components of MetS were associated with PSQI (all P values > .050). However, an increasing number of MetS components was associated with increasing probability of good quality of sleep (P for trend = .002), and of PSQI below the median (P for trend = .007). Generalized Additive Model analysis documented no smoothing function suggestive of nonlinear association between PSQI and MetS.

**Conclusion:**

Our results confirm a high prevalence of poor sleep quality in oldest age; however, in these subjects, MetS seems to be associated with better sleep quality. Additional larger, dedicated studies are required to confirm our results, and, if so, to identify the subsystems involved and the potential therapeutic implications of such an association.

## Introduction

Metabolic syndrome (MetS) is an intriguing entity, because it includes potentially reversible risk factors and is associated, at least in middle-aged populations, with several adverse outcomes, including cardiovascular and cerebrovascular morbidity and mortality [1].

On the other hand, several studies have indicated that sleep duration and architecture are associated with obesity, diabetes, hypertension, cardiovascular disease, and in turn, with MetS [2–4]. Several data indicate an association between the characteristics of sleep and the risk of cardiovascular events. For instance, the duration of sleep has been associated with left ventricular function and structure [5], while sleep disruption has been linked with atrial fibrillation [6]. The quality of sleep maintains its prognostic value even in the oldest age. In fact, in oldest subjects a self-rated poor quality of sleep is associated with reduced survival [7], and an objective measure of sleep quality has been associated with physical performance [8].

In a meta-analysis including over 75,000 participants the probability of MetS increased by 16% for a sleep duration below 8 h [9], and in a prospective cohort study sleep restriction resulted a risk factor for incident MetS [10]. Indeed, sleep plays a key role in maintaining circadian rhythm and metabolic and hormonal homeostasis [11]. Not surprisingly, the impairment of quantity and quality of sleep represents a risk factors for cardio-metabolic diseases, depression and cognitive impairment [12]. Therefore, ensuring adequate sleep quality is essential for improving quality of life and reducing mortality [13, 14]. On the other hand, MetS has been proven an independent risk factor for respiratory disorders, that in turn affect quality of sleep [4]. The potential association of MetS with sleep quality is further confounded by several factors, including subclinical inflammation and nutritional status.

Indeed, MetS has not so far been associated with clinically meaningful outcomes in the very old, and in some studies it has even been associated with positive outcomes, including better cognitive functioning, higher hemoglobin levels, quality of life, and functional ability [15–19]. Specifically, an association between poor sleep quality and MetS has been reported in younger and middle-aged adults; however, data are lacking regarding older and oldest populations [2, 3]. Thus, the aims of the present study were to assess the association, if any, of MetS with quality of sleep in a cohort of oldest subjects, and whether the direction of such an association is the same observed in younger populations.

## Methods

### Population

This study is based upon data from the “Mugello Study”, a survey of nonagenarians (i.e., subjects aged 90+) living in the Mugello area, north-eastward of Florence, in Tuscany, Italy. Details on aims, design and methods of the study are reported elsewhere [20]. Briefly, 475 unselected nonagenarians underwent structured interview and medical visit at home, along with comprehensive instrumental assessment and collection of blood samples. The Mugello Study protocol, which complied with the principles of the Declaration of Helsinki on clinical research involving human beings, was approved by the Institutional Review Board; participants, or their proxies, signed the informed consent form.

Data for the present study were available for 343 subjects, as we excluded from the original sample 91 participants with diagnosis of dementia, and 41 because of missing data for the study variables.

### Sleep quality

The Pittsburgh Sleep Quality Index (PSQI) was adopted to test the quality and pattern of sleep over the preceding 4 weeks [21]. The PSQI differentiated “poor” from “good” sleep by measuring the following seven areas: subjective sleep quality, sleep latency, sleep duration, habitual sleep efficiency, sleep disturbances, use of hypnotics, and daytime dysfunction. Scoring was based on a 0–3 Likert scale, and the sum of scores for these seven components yielded a global score ranging 0 to 21. A total score ≥ 5 represents inappropriate sleep quality, higher scores indicating lower quality of sleep. Using a cut-off score of 5, the PSQI has a sensitivity of 89.6% and specificity of 86.5% for identifying cases with a sleep disorder [22, 23].

### Metabolic syndrome

MetS was defined according to the National Cholesterol Education Program’s ATP-III criteria, adding use of hypolipemic, hypoglycemic, and antihypertensive medications as in several previous epidemiological studies [24]. The diagnosis of the MetS was defined by the presence of three or more of the following features: waist circumference > 88 cm in women and > 102 cm in men; fasting serum triglycerides ≥ 150 mg/dL; serum HDL < 50 mg/dL in women and < 40 mg/dL in men, or use of hypolipemic drugs; blood pressure ≥ 130/85 mmHg, or use of antihypertensive drugs; fasting blood glucose levels ≥ 110 mg/dL, or use of hypoglycaemic drugs.

### Covariates

Education was expressed as years of school attendance. Comorbidity was quantified using the Charlson score [25]. Body Mass Index (BMI) was calculated as weight (kg) divided by height squared (m^2^). Functional ability was estimated using the Katz’ activities of daily living (ADLs) [26] and the Lawton and Brody scale for instrumental activities of daily living (IADLs) [27]. Disability in the ADLs was defined as need of assistance for performing two or more ADLs. The reason for not choosing a single-point decline is that impairment in two ADLs is less likely to capture physiological fluctuations in functional performance. Impairment in IADL function was identified by a score < 7; this higher cut-off level is generally adopted to avoid a “floor effect”. Cognition was assessed using the Mini Mental State Examination [28]. Adherence to a Mediterranean-style diet was computed using the Mediterranean Diet Score [29]. Physical performance was assessed using the Short Physical Performance Battery, which includes three tests of physical function: balance, gait speed, and chair stands [30]. Drugs were coded according to the Anatomical Therapeutic and Chemical codes/Defined Daily Dose [31]. Diagnoses were coded according to the International Classification of Diseases, ninth edition, Clinical Modification codes [32]. Glomerular Filtration Rate was estimated using the Cockcroft-Gault equation.

### Statistical analysis

Statistical analyses were performed using SPSS for Mac 20.0, and R-3.5.3. Differences were considered significant at the P < .050 level. Data of continuous variables are presented as mean values ± standard deviation (SD). Median and inter-quartile ranges were provided for non-normally distributed variables. Analysis of variance (ANOVA) for normally distributed variables was performed according to good quality of sleep (i.e. PSQI < 5), and MetS; otherwise, the nonparametric Mann–Whitney U test was adopted. The two-tailed Fisher exact test was used for dichotomous variables. The covariates to be included into multivariable analyses were chosen as explanatory according to available evidence from the electronic databases of PubMed (MEDLINE) and Cochrane Library, and based upon biological plausibility. All the variables associated with either the PSQI or the MetS were included among the covariates in multivariable analyses [33].

Multivariable linear regression analysis was adopted to estimate the association of PSQI with age, sex, MetS, and all those variables which differed significantly (P < .05) in univariable analyses (Tables 1, and 2).

**Table 1:** Characteristics of 343 participants according to a Pittsburgh Sleep Quality Assessment index (PSQI) < 5 (indicating good quality of sleep)

	Participants with PSQI < 5 (n = 83)	Participants with PSQI ≥ 5 (n = 260)	P
N (%), mean (SD) or median (interquartile range) [traditional units]			
Demographics and lifestyle habits			
Age (years)	92 (3)	93 (3)	.455
Sex (female)	54 (65)	182 (70)	.816
Education (years)	5 (3–5)	4 (3–5)	.404
Alcohol consumption (mL/day)	4.5 (.9)	4.7 (.7)	.169
Smoking (total lifetime pack years)	3650 (1825–7300)	3650 (1460–7300)	.676
Mediterranean diet score	34.9 (2.9)	34.1 (3.8)	.081
Living in nursing home	8 (10)	21 (8)	.653
Medications			
ACE-inhibitors	31 (37)	93 (36)	.896
Diuretics	43 (52)	137 (53)	.900
Beta-blockers	16 (20)	37 (14)	.293
Platelet antiaggregants	46 (55)	90 (35)	.001
Benzodiazepines	7 (8)	56 (22)	.006
Antidepressants	14 (17)	47 (18)	.870
Antipsychotics	4 (5)	22 (8)	.346
Analgesics or oppioids	4 (5)	22 (8)	.346
Non steroidal anti-inflammatory drugs	7 (8)	40 (15)	.142
Corticosteroids	1 (1)	16 (6)	.083
Antihistaminics	0	1 (1)	.999
Comorbid conditions			
Charlson comorbidity Index	1 (0–3)	1 (0–4)	.440
Metabolic syndrome	28 (34)	47 (18)	.004
Heart failure	12 (15)	67 (27)	.035
Chronic pulmonary disease	10 (13)	38 (15)	.715
Stroke or cerebrovascular disease	12 (15)	55 (22)	.203
Peripheral arterial disease	16 (20)	50 (20)	.999
Connectivities	3 (4)	5 (2)	.409
Hepatic disease	4 (5)	10 (4)	.751
Depression	1 (1)	15 (6)	.132
Physical and cognitive performance			
Mini mental state examination	20.1 (9.2)	21.9 (6.7)	.052
Disability in BADL	33 (40)	129 (49)	.165
Disability in IADL	69 (83)	225 (86)	.472
Short physical performance battery	7 (3)	6 (3)	.225
Body mass index (Kg/m^2^)	25.5 (4.2)	25.5 (4.9)	.972
Biohumoral parameters			
Glomerular filtration rate (mL/min)	39.5 (16.7)	40.1 (16.9)	.800
Hemoglobin (g/L) [g/dL)]	131 (17) [13.1 (1.7)]	128 (15) [12.8 (1.5)]	.187
Total serum proteins (g/dL) [g/L]	65 (7) [6.5 (.7)]	65 (6) [6.5 (.6)]	.536
Albumin (proportion of 1) [%]	.56 (.05) [55.6 (4.6)]	.57 (.04) [57.2 (4.4)]	.013
C-reactive protein (nmol/L) [mg/L]	3.43 (1.71–6.95) [.36 (.18–.73)]	3.90 (1.62–7.52) [.41 (.17–.79)]	.347

**Table 2:** Characteristics of 343 participants according to the presence of metabolic syndrome

	Participants with metabolic syndrome (n = 75)	Participants without metabolic syndrome (n = 268)	P
N (%), mean (SD) or median (interquartile range) [traditional units]			
Demographics and lifestyle habits			
Age (years)	92 (3)	93 (3)	.028
Sex (female)	59 (79)	177 (66)	.048
Education (years)	5 (3–5)	5 (3–6)	.776
Alcohol consumption (mL/day)	4.7 (.7)	4.6 (.8)	.769
Smoking (total lifetime pack years)	4867 (3650–7300)	3650 (1217–7300)	.071
Mediterranean diet score	34.0 (4.0)	34.3 (3.5)	.526
Living in nursing home	7 (9)	22 (8)	.814
Medications			
ACE-inhibitors	35 (47)	89 (33)	.041
Diuretics	39 (52)	141 (53)	.999
Beta-blockers	19 (26)	34 (13)	.010
Platelet antiaggregants	32 (43)	104 (39)	.594
Benzodiazepines	9 (12)	54 (20)	.129
Antidepressants	19 (25)	42 (16)	.062
Antipsychotics	5 (7)	21 (8)	.999
Analgesics or oppioids	6 (8)	20 (7)	.810
Non steroidal anti-inflammatory drugs	13 (17)	34 (13)	.343
Corticosteroids	4 (5)	13 (5)	.772
Antihistaminics	0 (0)	1 (.4)	.999
Comorbid conditions			
Charlson comorbidity Index	1 (0–6)	1 (0–3)	.035
Heart failure	13 (18)	66 (26)	.166
Chronic pulmonary disease	10 (14)	38 (15)	.853
Stroke or cerebrovascular disease	16 (22)	51 (20)	.745
Peripheral arterial disease	13 (18)	53 (21)	.662
Connectivities	1 (1)	7 (3)	.689
Hepatic disease	3 (4)	2 (1)	.077
Depression	2 (3)	14 (5)	.538
Physical and cognitive performance			
Mini mental state examination	21.2 (8.0)	21.6 (7.2)	.745
Disability in BADL	34 (45)	127 (47)	.794
Disability in IADL	67 (89)	227 (85)	.356
Short physical performance battery	6 (2)	6 (3)	.676
Body mass index (kg/m^2^)	27.4 (4.1)	24.9 (4.8)	<.0001
Pittsburgh sleep quality assessment	6 (4)	8 (4)	.007
Biohumoral parameters			
Glomerular filtration rate (mL/min)	37.2 (16.0)	40.0 (17.0)	.121
Hemoglobin (g/L) [g/dL]	124 (16) [12.4 (1.6)]	130 (15) [13.0 (1.5)]	.002
Total serum proteins (g/dL) [g/L]	64 (7) [6.4 (.7)]	65 (6) [6.5 (.6)]	.371
Albumin (proportion of 1) [%]	.57 (.05) [56.9 (4.8)]	.58 (.04) [56.7 (4.4)]	.811
C-reactive protein (nmol/L) [mg/L]	3.43 (1.52–7.43) [.44 (.16–.78)]	2.67 (1.43–6.67) [.28 (.15–.70)]	.302

Multivariable logistic regression analysis was adopted to estimate the association of a good quality of sleep (i.e. PSQI < 5) with age, sex, MetS, and all those variables which differed significantly (P < .05) in univariable analyses (Tables 1, and 2); the BMI was not entered because of its collinearity with MetS. The same set of covariates was also entered in logistic regression having a PSQI below the median value as dependent variable. To rule out any role of body size, logistic models were analyzed after the insertion of the BMI instead of MetS. Also, the same set of covariates was analyzed after the inclusion of the Mini Mental State Examination score in the multivariable model, to take into account the role of cognition in the association between sleep and MetS. The same multivariable regression models were analyzed after stratifying for sex. Analysis of the interaction term sex*MetS was performed to assess the role of gender in the association between MetS and quality of sleep.

Eventually, the multivariable logistic models were analyzed after entering the single items of Mets, as well as the number of MetS components.

Collinearity diagnostics estimated by the Variance Inflation Factor indicated no collinearity between the covariates entered into the models (all VIF values < 2); also, test of goodness of fit performed by the Hosmer and Lemeshow Test indicated a good fit of the models (all P values > .050).

Eventually, generalized additive model (GAM) analysis was performed to rule out any curvilinear relationship of metabolic syndrome with quality of sleep.

Based upon available data, [34] analysis by G*Power indicated that a sample of 302 elderly patients would allow over 80% power for detecting a 1.9 OR in logistic regression analysis (α = .05) having a good quality of sleep as the dependent outcome.

## Results

In our population, good quality of sleep was reported by 83 (24%) subjects, while 178 (52%) subjects had a PSQI score ≤ the median value; the median PSQI was 7 (5–10). MetS was diagnosed in 75 (22%) participants. Participants with MetS had a significantly lower PSQI than subjects without MetS (6 (3–10) vs 8 (5–11); P = .005).

The main characteristics of the population according to a PSQI < 5 are depicted in Table 1. Specifically, as compared with controls, they had less prevalent diagnosis of heart failure, reported a less prevalent use of benzodiazepines, and a more prevalent use of antiplatelets; also, they showed lower serum albumin levels; eventually, participants with a good quality of sleep had a more prevalent diagnosis of MetS.

The main characteristics of the population according to the presence of metabolic syndrome are depicted in Table 2. Specifically, as compared with controls, they were more likely younger females with higher body mass index; they had an higher Charlson scores; also, they reported a more prevalent use of ACE-inhibitors and beta-blockers; they showed lower hemoglobin levels; eventually, participants with MetS reported a lower PSQI score, as compared with controls.

In linear regression, PSQI was inversely associated with MetS in the crude model (B = -1.33; 95% CI − 2.28 to − .37; P = .007), after adjusting for age and sex (B = − 1.36; 95% CI − 2.33 to − .39; P = .006), and in the multivariable model (B = − 1.04; 95% CI − 2.06 to − .03; P = .044; Table 3).

**Table 3:** Association of the Pittsburgh sleep quality assessment index (PSQI) with the variables of interest

	Linear regression model		
	PSQI as a continuous variable		
	B	95% CI	P
Age (each year)	− .05	− .20 to .09	.467
Sex (female)	.22	− .77 to 1.22	.658
Benzodiazepines	1.44	.26 to 2.61	.017
Platelet antiaggregants	− 1.84	− 2.76 to − .92	.000
ACE-inhibitors	− .14	− 1.25 to .97	.800
Beta-blockers	.27	− 1.02 to 1.56	.683
Charlson comorbidity Index	.07	− .13 to .27	.495
Heart failure	.92	− .16 to 2.00	.095
Albumin (proportion of 1) [%]	− .38	− 1.11 to .34	.296
Hemoglobin (g/L) [g/dL]	− .12	− .42 to .19	.443
Metabolic syndrome	− 1.04	− 2.06 to − .03	.044

	Logistic regression model		
	PSQI < 5		
	OR	95% CI	P
Age (each year)	1.01	.91 to 1.12	.856
Sex (female)	1.03	.54 to 1.99	.920
Benzodiazepines	.31	.12 to .80	.015
Platelet antiaggregants	2.25	1.22 to 4.17	.010
ACE-inhibitors	.88	.47 to 1.67	.698
Beta-blockers	1.02	.46 to 2.29	.953
Charlson comorbidity Index	.92	.80 to 1.07	.276
Heart failure	.49	.23 to 1.08	.076
Albumin (proportion of 1) [%]	.91	.85 to .98	.012
Hemoglobin (g/L) [g/dL]	1.17	.95 to 1.44	.139
Metabolic syndrome	2.52	1.26 to 5.02	.009

	Logistic regression model		
	PSQI ≤ 7		
	OR	95% CI	P
Age (each year)	.97	.89 to 1.06	.536
Sex (female)	.60	.33 to 1.06	.079
Benzodiazepines	.40	.20 to .81	.011
Platelet antiaggregants	1.85	1.08 to 3.16	.025
ACE-inhibitors	1.81	1.04 to 3.15	.037
Beta-blockers	.93	.44 to 1.94	.839
Charlson comorbidity Index	.95	.84 to 1.07	.424
Heart failure	.89	.47 to 1.66	.705
Albumin (proportion of 1) [%]	.95	.89 to 1.04	.069
Hemoglobin (g/L) [g/dL]	1.08	.90 to 1.30	.413
Metabolic syndrome	2.11	1.11 to 3.40	.022

All the covariates were entered simultaneously into the regression models. The model included all the variables which differed significantly (P < .050) in univariable analyses in Tables 1, and 2

In logistic regression a good quality of sleep (i.e. PSQI < 5) was associated with MetS in the crude model (OR = 2.31; 95% CI 1.33–4.01; P = .003), after adjusting for age and sex (OR = 2.40; 95% CI 1.36–4.23; P = .003), as well as in the multivariable model (OR = 2.52; 95% CI 1.26–5.02; P = .009; Table 3).

Likewise, MetS was associated with increased probability of a PSQI below the median (i.e. PSQI ≤ 7) in the crude model (OR = 2.19; 95% CI 1.28–3.74), after adjusting for age and sex (OR = 2.26; 95% CI 1.30–3.91; P = .004), and in the multivariable model (OR = 2.11; 95% CI 1.1–3.40, P = .022 Table 3).

In contrast, no association was found (B = .01; 95% CI − .08 to .11; P = .772 for PSQI as a continuous variable; OR = 1.01; 95% CI .95–1.07; P = .847 for PSQI < 5, and OR = 1.01; 95% CI .95–1.06; P = .912 for PSQI ≤ 7) for BMI when it was entered instead of MetS in the multivariable models.

Furthermore, after adjusting for cognitive performance, although in multivariable linear model metabolic syndrome was not significantly associated with the whole spectrum of the PSQI (B = − .86; 95% CI − 1.86 to.14; P = .092), in logistic multivariable models MetS was associated with a good quality of sleep (OR = 2.42; 95% CI 1.20–4.88, P = .014) and with PSQI below the median (OR = 2.06; 95% CI 1.09–3.92, P = .027).

When the multivariable regression models were analyzed after stratifying for sex, MetS was statistically associated with the PSQI score (B = − 2.66, 95% CI − 4.51 to .81; P = .005), with a good quality of sleep (OR = 4.64; 95% CI 1.20–18.00, P = .026), and with a PSQI below the median (OR = 12.93; 95% CI 2.26–23.98, P = .004) in men; in women MetS was not significantly associated with the PSQI score (B − .72, 95% CI − 1.94 to .50; P = .247); however a significant association was found of MetS with a good quality of sleep (OR = 2.70; 95% CI 1.22–6.01, P = .015), and with a PSQI below the median (OR = 2.09; 95% CI 1.02–4.25, P = .043). Analysis of the interaction term confirmed that the association between quality of sleep and MetS was not affected by sex (P = .215 for PSQI; P = . 532 for PSQI < 5; P = .724 for PSQI below the median).

None of the single components of MetS was significantly associated with a good quality of sleep or with a PSQI below the median (Table 4), also after stratifying for sex. However, an increasing number of MetS components was associated with increasing probability of good quality of sleep (P for linear trend = .005), as well as of a PSQI ≤ 7 (P for linear trend = .024), after adjusting.

**Table 4:** Association of Pittsburgh sleep quality assessment index (PSQI) with the single components of MetS

	Linear regression model		
	PSQI as a continuous variable		
	B	95% CI	P
Abdominal obesity	− .24	− 1.30 to .81	.651
Hypertriglyceridemia	− 1.21	− 2.55 to .12	.074
Low HDL − cholesterol	.11	− 1.09 to 1.31	.855
High blood pressure	.07	− 1.05 to 1.19	901
High fasting blood glucose	− .23	− 1.43 to .96	.700

	Logistic regression model		
	PSQI < 5		
	OR	95% CI	P
Abdominal obesity	1.36	.72 to 2.57	.339
Hypertriglyceridemia	1.98	.94 to 4.15	.071
Low HDL-cholesterol	1.02	.50 to 2.07	.964
High blood pressure	.78	.41 to 1.48	.451
High fasting blood glucose	1.35	.67 to 2.71	.403

	Logistic regression model		
	PSQI ≤ 7		
	OR	95% CI	P
Abdominal obesity	1.41	.81 to 2.43	.222
Hypertriglyceridemia	1.60	.79 to 3.22	.190
Low HDL-cholesterol	.92	.49 to 1.71	.791
High blood pressure	1.53	.88 to 2.67	.132
High fasting blood glucose	1.46	.78 to 2.72	.239

All the covariates were entered simultaneously into the regression models. The model included all the variables which differed significantly (P < .050) in univariable analyses in Tables 1, and 2 (i.e. age, sex, use of benzodiazepines, platelet antiaggregants, ACE-inhibitors, and beta-blockers, diagnosis of heart failure, Charlson comorbidity score index, albumin, and hemoglobin levels)

Results of GAM analysis were summarized in Additional file 1: Table S1; overall, there was no smoothing function suggestive of nonlinear association between MetS and PSQI; however, when the single components of MetS were analyzed, there was a nonsignificant smoothed association of low HDL-cholesterol and hypertension with PSQI (Additional file 1: Table S1).

## Discussion

Our results indicate that MetS was independently associated with a better quality of sleep in our sample of community-dwelling oldest subjects; the syndrome in itself, rather than its individual components, seemed to be associated with sleep quality, with a linear association between increasing number of items and increasing probability of a better sleep quality. Our results confirmed data indicating that MetS and sleep seem associated and, in particular, lower night sleep hours are related to higher risk of development of MetS. This is particular shown for young and middle-aged people [35].

This finding is intriguing, because it is at variance with the common opinion that MetS might represent either a consequence or a cause of disturbed sleep. A possible explanation for our contrasting results is that previous studies which analyzed the correlation between MetS and sleep included much younger subjects [2–4, 9]. Of notice, MetS has been associated with better cognitive performance [16] and quality of life among subjects aged 80+ [17]; also, it has been linked with better functional ability in participants aged 75+ [18], while it is not a risk factor for reduced survival in the elderly, as in younger subjects [36]. Indeed, an association of MetS with a better quality of sleep should be expected in the light of these findings.

Interestingly, Qian and colleagues showed this relationship in relatively old adults [37]. Actually, they evaluated subjects aged at least 60 years old and found that the lowest risk of MetS is present in people who sleep for at least 7 h in the night. Additionally, even the day portion of sleeping is important. In fact the daytime napping may represent a non-functional sleep and increase the risk of MetS [37]. However, in comparison to our work, in Quian’s study people with a wide age range were considered and this should explain the difference with our results. Other studies found similar results but, usually, the age range is very variable and a substantial rarity of specific results for healthy nonagerians is visible [9, 38]. These last considerations is supported by the findings of other studies, where a wider assessment of the relationship with the age was performed. Stefani and colleagues evaluated Korean people and found that the very low sleep duration was correlated with MetS in middle-age men and they conclude that age and sex change the relationship between sleep duration and MetS [39]. This strengthens the necessary division of the population, on the basis of the age, to adequately perform studies assessing metabolic condition.

This counterintuitive behavior of MetS might belong to so-called “reverse epidemiology”, according to which factors improving short-term survival and/or well-being, albeit reducing long-term survival, are associated with decreased mortality and event rates among subjects with reduced life expectancy [40]. Thus, established risk factors in general populations yield paradoxically opposite predictive patterns in populations characterized by “frailty”. In these subjects the components of MetS reflect the absence of more powerful risk factors, including malnutrition, that most commonly, and in a shorter term, might affect health status and survival [40]. In this setting, our finding of an association between higher serum albumin levels and better quality of sleep confirm the role of a better nutritional status on the maintenance of sleep quality. Of notice, our results confirm a greater prevalence of heart failure in subjects with worse quality of sleep; obesity, increased serum cholesterol levels and higher blood pressure have been associated with reduced morbidity and mortality in subjects with frailty due to heart failure or end-stage renal disease, as well as in older subjects [41]. Also, the “obesity paradox” might allow to interprete our results. In fact, obesity has been associated with a better quality of life, and quality of sleep is a part of quality of life. Also, higher waist circunference has been associated with lower incidence of disability in people aged 90+ [42]. People with low weight may suffer from malnutrition, which makes them more susceptible to disease and disability [43], and malnutrition has been associated with poorer quality of sleep [44]. Moreover, quality of sleep is inversely related with sarcopenia and frailty [45].

Also, poor sleep quality could be a consequence of obstructive sleep apnea, which may derive from MetS [46]; thus, sleep apnea would be a mediator of this association, at least in younger population, while it has been showed that in an aged animal model, chronic intermittent hypoxia does not alter carotid body responses, catecholamine‐related parameters or redox status, and does not cause hypertension, thus authors argue that age affords protection to harmful effects produced by chronic intermittent hypoxia [47].

Furthermore, we have to consider that subjects who “survived” over 90 years despite MetS were probably less susceptible to its cardiovascular effects. Also, inflammation and oxidative stress are considered other mechanisms which link poor sleep with cardiometabolic risk profile in adults. Indeed, in sleep disordered populations elevated levels of inflammation and oxidative stress are common features [48]. However, this observation has not been confirmed in our population, as C Reactive Protein levels were not associated with quality of sleep. Eventually, it has been hypothesized that the common pathophysiological pathway of MetS and poor sleep is the hyperactivation of the hypothalamic-pituitary- adrenal axis [49]; indeed, such hyperactivation might account for an increased cardiovascular risk. However, such hyperactivation has been found in young subjects, but not confirmed in the elderly [50]. The association of good quality of sleep with MetS did not differ between men and women, as confirmed by the lack of interaction between MetS and sex.

### Strengths and limitations

The present study has some limitations. First, due to its cross-sectional design this study does not allow to ascertain any cause-effect relationship. Second, sleep was evaluated using the self-reported PSQI questionnaire, while no objective measure of sleep disturbance (e.g. polysomnography) was performed; however, subjective sleep quality represents a good prognostic marker even in very old populations [7]; in addition, there was lack of information about other indirect aspects of sleep quality, such as daytime sleepiness, which is explored by the Epworth Sleepness Scale. Eventually, only the overall score was available for the present analyses.

This study has also some strengths. First, to our knowledge this is the first study that analyzed the association between MetS and sleep in a population on nonagenarians. Second, we enrolled a community-dwelling population, with extensive information regarding risk factors, comorbid conditions, and objective parameters. Finally, as it included approximately 65% of nonagenarians living in the Mugello area, this sample seems to be fairly representative.

## Conclusion

In oldest subjects, MetS might be significantly associated with greater quality of sleep. Results of the present study might query the opportunity of treating MetS to improve quality of sleep or, conversely, of increasing sleep to reduce MetS prevalence in very old subjects. Together with other counterintuitive associations in very old populations, our results might caution against considering the evidence pertaining to adults and “young elderly” also valid for the “old old”. As the “old old” segment of general populations is dramatically rising, and not only in western countries, epidemiological surveys should pay more attention to this population, and clinical practice should capitalize on geriatric epidemiological research. Finally, longitudinal studies are still needed to confirm our findings.

## Supplementary information

**Additional file 1: Table S1.** Results of the generalized additive model having the metabolic syndrome and its single components as the dependent variable and quality of sleep as the independent variable.

## Data Availability

Not applicable.

## Abbreviation

MetS: Metabolic syndrome
PSQI: Pittsburgh sleep quality assessment index
BMI: Body mass index
ADLs: Activities of daily living
IADLs: Instrumental activities of daily living

## References

[1] Obunai K, Jani S, Dangas GD (2007). Cardiovascular morbidity and mortality of the metabolic syndrome. Med Clin North Am.

[2] Zohal M, Ghorbani A, Esmailzadehha N, Ziaee A, Mohammadi Z (2017). Association of sleep quality components and wake time with metabolic syndrome: the Qazvin Metabolic Diseases Study (QMDS), Iran. Diabetes Metab Syndr.

[3] Lin S-C, Sun C-A, You S-L, Hwang L-C, Liang C-Y, Yang T (2016). The link of self-reported insomnia symptoms and sleep duration with metabolic syndrome: a Chinese population-based study. Sleep.

[4] Baffi CW, Wood L, Winnica D, Strollo PJ, Gladwin MT, Que LG (2016). Metabolic syndrome and the lung. Chest.

[5] Lee J-H, Park SK, Ryoo J-H, Oh C-M, Kang JG, Mansur RB (2018). Sleep duration and quality as related to left ventricular structure and function. Psychosom Med.

[6] Christensen MA, Dixit S, Dewland TA, Whitman IR, Nah G, Vittinghoff E (2018). Sleep characteristics that predict atrial fibrillation. Heart Rhythm.

[7] Qiu L, Sautter J, Liu Y, Gu D (2011). Age and gender differences in linkages of sleep with subsequent mortality and health among very old Chinese. Sleep Med.

[8] Kim M, Yoshida H, Sasai H, Kojima N, Kim H (2015). Association between objectively measured sleep quality and physical function among community-dwelling oldest old Japanese: a cross-sectional study. Geriatr Gerontol Int.

[9] Iftikhar IH, Donley MA, Mindel J, Pleister A, Soriano S, Magalang UJ (2015). Sleep duration and metabolic syndrome. An updated dose-risk metaanalysis. Ann Am Thorac Soc.

[10] Venancio DP, Suchecki D (2015). Prolonged REM sleep restriction induces metabolic syndrome-related changes: mediation by pro-inflammatory cytokines. Brain Behav Immun.

[11] Sollars PJ, Pickard GE (2015). The neurobiology of circadian rhythms. Psychiatr Clin North Am.

[12] Reutrakul S, Knutson KL (2015). Consequences of circadian disruption on cardiometabolic health. Sleep Med Clin.

[13] Maggi S, Langlois JA, Minicuci N, Grigoletto F, Pavan M, Foley DJ (1998). Sleep complaints in community-dwelling older persons: prevalence, associated factors, and reported causes. J Am Geriatr Soc.

[14] Bellia V, Catalano F, Scichilone N, Incalzi RA, Spatafora M, Vergani C (2003). Sleep disorders in the elderly with and without chronic airflow obstruction: the SARA study. Sleep.

[15] Laudisio A, Bandinelli S, Gemma A, Ferrucci L, Antonelli Incalzi R (2013). Metabolic syndrome and hemoglobin levels in elderly adults: the Invecchiare in Chianti Study. J Am Geriatr Soc.

[16] Laudisio A, Marzetti E, Pagano F, Cocchi A, Franceschi C, Bernabei R (2008). Association of metabolic syndrome with cognitive function: the role of sex and age. Clin Nutr Edinb Scotl.

[17] Laudisio A, Marzetti E, Antonica L, Pagano F, Vetrano DL, Bernabei R (2013). Metabolic syndrome and quality of life in the elderly: age and gender differences. Eur J Nutr.

[18] Laudisio A, Bandinelli S, Gemma A, Ferrucci L, Incalzi RA (2014). Metabolic syndrome and functional ability in older age: the InCHIANTI study. Clin Nutr Edinb Scotl.

[19] Giovannini S, Tinelli G, Biscetti F, Straface G, Angelini F, Pitocco D (2017). Serum high mobility group box-1 and osteoprotegerin levels are associated with peripheral arterial disease and critical limb ischemia in type 2 diabetic subjects. Cardiovasc Diabetol.

[20] Molino-Lova R, Sofi F, Pasquini G, Gori A, Vannetti F, Abbate R (2013). The Mugello study, a survey of nonagenarians living in Tuscany: design, methods and participants’ general characteristics. Eur J Intern Med.

[21] Buysse DJ, Reynolds CF, Monk TH, Berman SR, Kupfer DJ (1989). The Pittsburgh Sleep Quality Index: a new instrument for psychiatric practice and research. Psychiatry Res.

[22] Buysse DJ, Hall ML, Strollo PJ, Kamarck TW, Owens J, Lee L (2008). Relationships between the Pittsburgh Sleep Quality Index (PSQI), Epworth Sleepiness Scale (ESS), and clinical/polysomnographic measures in a community sample. J Clin Sleep Med JCSM Off Publ Am Acad Sleep Med.

[23] Grandner MA, Kripke DF, Yoon I-Y, Youngstedt SD (2006). Criterion validity of the Pittsburgh sleep quality index: investigation in a non-clinical sample. Sleep Biol Rhythms.

[24] Grundy SM, Brewer HB, Cleeman JI, Smith SC, Lenfant C, American Heart Association (2004). Definition of metabolic syndrome: report of the National Heart, Lung, and Blood Institute/American Heart Association conference on scientific issues related to definition. Circulation.

[25] Charlson ME, Pompei P, Ales KL, MacKenzie CR (1987). A new method of classifying prognostic comorbidity in longitudinal studies: development and validation. J Chronic Dis.

[26] Katz S, Ford AB, Moskowitz RW, Jackson BA, Jaffe MW (1963). Studies of illness in the aged. The index of ADL: a standardized measure of biological and psychosocial function. JAMA.

[27] Lawton MP, Brody EM (1969). Assessment of older people: self-maintaining and instrumental activities of daily living. Gerontologist.

[28] Folstein MF, Folstein SE, McHugh PR (1975). “Mini-mental state”. A practical method for grading the cognitive state of patients for the clinician. J Psychiatr Res.

[29] Trichopoulou A, Costacou T, Bamia C, Trichopoulos D (2003). Adherence to a mediterranean diet and survival in a Greek population. N Engl J Med.

[30] Guralnik JM, Simonsick EM, Ferrucci L, Glynn RJ, Berkman LF, Blazer DG (1994). A short physical performance battery assessing lower extremity function: association with self-reported disability and prediction of mortality and nursing home admission. J Gerontol.

[31] http://www.whocc.no/atc_ddd_index/ n.d.

[32] WHO | International Classification of Diseases. WHO n.d. http://www.who.int/classifications/icd/en/. Accessed 11 Oct 2017.

[33] Skelly AC, Dettori JR, Brodt ED (2012). Assessing bias: the importance of considering confounding. Evid-Based Spine-Care J.

[34] Lim YC, Hoe VCW, Darus A, Bhoo-Pathy N (2018). Association between night-shift work, sleep quality and metabolic syndrome. Occup Environ Med.

[35] Chaput J-P, McNeil J, Després J-P, Bouchard C, Tremblay A (2013). Seven to eight hours of sleep a night is associated with a lower prevalence of the metabolic syndrome and reduced overall cardiometabolic risk in adults. PLoS ONE.

[36] Hao Q, Song X, Yang M, Dong B, Rockwood K (2016). Understanding risk in the oldest old: frailty and the metabolic syndrome in a chinese community sample aged 90+ years. J Nutr Health Aging.

[37] Qian Y-X, Liu J-H, Ma Q-H, Sun H-P, Xu Y, Pan C-W (2019). Associations of sleep durations and sleep-related parameters with metabolic syndrome among older Chinese adults. Endocrine.

[38] Smiley A, King D, Bidulescu A (2019). The association between sleep duration and metabolic syndrome: the NHANES 2013/2014. Nutrients.

[39] Stefani KM, Kim HC, Kim J, Oh K, Suh I (2013). The influence of sex and age on the relationship between sleep duration and metabolic syndrome in Korean adults. Diabetes Res Clin Pract.

[40] Ahmadi S-F, Streja E, Zahmatkesh G, Streja D, Kashyap M, Moradi H (2015). Reverse epidemiology of traditional cardiovascular risk factors in the geriatric population. J Am Med Dir Assoc.

[41] Kalantar-Zadeh K, Block G, Horwich T, Fonarow GC (2004). Reverse epidemiology of conventional cardiovascular risk factors in patients with chronic heart failure. J Am Coll Cardiol.

[42] Lisko I, Tiainen K, Raitanen J, Jylhävä J, Hurme M, Hervonen A (2017). body mass index and waist circumference as predictors of disability in nonagenarians: the vitality 90+ study. J Gerontol A Biol Sci Med Sci.

[43] Ahmed T, Haboubi N (2010). Assessment and management of nutrition in older people and its importance to health. Clin Interv Aging.

[44] Şengül Ş, Uysal H (2019). The relationship between the nutritional status and sleep quality of patients with atrial fibrillation. Saudi Med J.

[45] Tuna F, Üstündağ A, Başak Can H, Tuna H (2019). Rapid geriatric assessment, physical activity, and sleep quality in adults aged more than 65 years: a preliminary study. J Nutr Health Aging.

[46] Turino C, Bertran S, Gavaldá R, Teixidó I, Woehrle H, Rué M (2017). Characterization of the CPAP-treated patient population in Catalonia. PLoS ONE.

[47] Quintero M, Olea E, Conde SV, Obeso A, Gallego-Martin T, Gonzalez C (2016). Age protects from harmful effects produced by chronic intermittent hypoxia. J Physiol.

[48] Kim J, Yoon DW, Lee SK, Lee S, Choi K-M, Robert TJ (2017). Concurrent presence of inflammation and obstructive sleep apnea exacerbates the risk of metabolic syndrome: a KoGES 6-year follow-up study. Medicine (Baltimore).

[49] Gaffey AE, Bergeman CS, Clark LA, Wirth MM (2016). Aging and the HPA axis: stress and resilience in older adults. Neurosci Biobehav Rev.

[50] van Neijenhof RJGP, van Duijn E, Van Den Berg JF, de Waal MWM, van der Mast RC, Comijs HC (2017). Subjective insomnia symptoms and sleep duration are not related to hypothalamic-pituitary-adrenal axis activity in older adults. J Sleep Res.

