# Supplementary material for: Metabolic syndrome is associated with better quality of sleep in the oldest old: results from the “Mugello Study”
Source: Diabetol Metab Syndr. 2020 May 24;12:46. doi: 10.1186/s13098-020-00554-y (PMC7386243; doi:10.1186/s13098-020-00554-y)
Supplement: Supplementary file 1 — Additional file 1: Table S1. Results of the generalized additive model having the metabolic syndrome and its single components as the dependent variable and quality of sleep as the independent variable. [file 13098_2020_554_MOESM1_ESM.docx]

Table S1. Results of the Generalized Additive Model having the metabolic syndrome and its single components as the dependent variable and quality of sleep as the independent variable*

|  | **Metabolic syndrome** | |
| --- | --- | --- |
|  | Effective Degree of Freedom | P |
| PSQI | 1.00 | .0012 |
|  | **Abdominal obesity** | |
|  | Effective Degree of Freedom | P |
| PSQI | 1.00 | .801 |
|  | **Hypertriglyceridemia** | |
|  | Effective Degree of Freedom | P |
| PSQI | 1.00 | .099 |
|  | **Low HDL−cholesterol** | |
|  | Effective Degree of Freedom | P |
| PSQI | 2.476 | .220 |
|  | **High blood pressure** | |
|  | Effective Degree of Freedom | P |
| PSQI | 1.725 | .608 |
|  | **High fasting blood glucose** | |
|  | Effective Degree of Freedom | P |
| PSQI | 1.00 | .093 |

* The model included all the variables which differed significantly (P<.050) in univariable analyses in Table 1, and 2 (i.e. age, sex, use of benzodiazepines, platelet antiaggregants, ACE-inhibitors, and beta-blockers, diagnosis of heart failure, Charlson comorbidity score index, albumin, and hemoglobin levels)
